# Claudin-18 expression in oesophagogastric adenocarcinomas: a tissue microarray study of 523 molecularly profiled cases

**DOI:** 10.1038/s41416-019-0508-4

**Published:** 2019-06-25

**Authors:** Irene Coati, Gábor Lotz, Giuseppe Nicolò Fanelli, Stefano Brignola, Cristiano Lanza, Rocco Cappellesso, Antonio Pellino, Salvatore Pucciarelli, Gaya Spolverato, Vincenza Guzzardo, Giada Munari, Giovanni Zaninotto, Marco Scarpa, Luca Mastracci, Fabio Farinati, Stefano Realdon, Pierluigi Pilati, Sara Lonardi, Nicola Valeri, Massimo Rugge, Andras Kiss, Fotios Loupakis, Matteo Fassan

**Affiliations:** 1 Department of Medicine (DIMED), Surgical Pathology & Cytopathology Unit, University of Padua, Padua, Italy; 2 2nd Department of Pathology, Semmelweis University, Budapest, Hungary; 3 Unit of Oncology 1, Department of Oncology, Veneto Institute of Oncology IOV – IRCCS, Padua, Italy; 4 Department of Surgical Oncology and Gastroenterology Sciences (DiSCOG), Surgery Unit, University of Padua, Padua, Italy; 5 Department of Surgery, Imperial College, London, UK; 6 Department of Surgical Sciences and Integrated Diagnostics (DISC), Pathology Unit, University of Genova, Genova, Italy; 7 Department of Surgical Oncology and Gastroenterology Sciences (DiSCOG), Gastroenterology Unit, University of Padua, Padua, Italy; 8 Unit of Gastroenterology, Veneto Institute of Oncology IOV – IRCCS, Padua, Italy; 9 Unit of Surgical Oncology of the Esophagus and Digestive Tract, Veneto Institute of Oncology IOV – IRCCS, Padua, Italy; 10 Division of Molecular Pathology, Institute of Cancer Research, London, UK; 11 Department of Medicine, Royal Marsden Hospital, London, UK; 12 Veneto Cancer Registry, Padua, Italy

**Keywords:** Gastric cancer, Molecular medicine

## Abstract

**Background:**

Claudin-18 (CLDN18) is a highly specific tight junction protein of the gastric mucosa. An isoform of CLDN18, the Claudin 18.2, has recently emerged as an innovative drug target for metastatic gastric cancer.

**Methods:**

We investigated the immunohistochemical profile of CLDN18, p53, p16, E-cadherin, MSH2, MSH6, MLH1, PSM2, HER2, and PDL-1 in a large series of 523 primary gastric carcinomas (GCs; *n* = 408) and gastro-oesophageal carcinomas (GECs; *n* = 115) and 135 matched and synchronous nodal metastases. The status of HER2 and EBER by means of chromogenic in situ hybridisation (CISH) was also evaluated.

**Results:**

High membranous CLDN18 expression was present in 150/510 (29.4%) primary cases and in 45/132 (34.1%) metastases. An abnormal expression (i.e. nuclear and/or cytoplasmic) was observed in 115 (22.5%) primary cases and in 33 (25.0%) metastases. A 38.8% of the cases showed significant CLDN18 intratumoural variability among the different tissue microarray cores obtained from the same tumour. Positive membrane CLDN18 expression was statistically associated with non-antral GCs (*p* = 0.016), Lauren diffuse type (*p* = 0.009), and with EBV-associated cancers (*p* < 0.001).

**Conclusions:**

CLDN18 is frequently expressed in gastric and gastro-oesophageal cancers; further studies should investigate the prognostic significance of CLDN18 heterogeneity in order to implement its test into clinical practice.

## Background

Gastric (GCs) and gastro-oesophageal (GECs) carcinomas are the third leading cause of cancer-related death world-wide with a combined incidence of 1.4 million cases annually.^1^

In 2014, The Cancer Genome Atlas (TCGA) project proposed a molecular classification of gastric cancer, dividing it into four subtypes: Epstein–Barr virus (EBV)-associated cancers, microsatellite instable (MSI) tumours, genomically stable (GS) tumours, and tumours with chromosomal instability (CIN).^2^ The worldwide prevalence of EBV-associated GC is about 5–25%.^3^ The clinicopathological features of EBV-associated GCs include a male predominance, a typical proximal (non-antral) localisation, a moderate-to-poor differentiation and a lymphocytic infiltration.^3,4^ The clinicopathological characteristics of MSI GCs include older age, lower frequency of nodal involvement, better prognosis, high cellular pleomorphism and higher prevalence of tumour-associated inflammatory infiltration.^5^ Genomically stable (GS) GCs and tumours with CIN have no specific clinicopathological features, even if the first mostly shows a diffuse pattern and the second is often located at the gastro-oesophageal junction.

Each subtype shows a peculiar molecular background.^2^ EBV-associated neoplasms usually have the cyclin-dependent kinase inhibitor 2A gene (*CDKN2A*) silencing, the programmed death-ligand 1 (PD-L1) overexpression and mutations in phosphatidylinositol-4,5-bisphosphate 3-kinase catalytic subunit alpha (*PIK3CA*). MSI tumours are characterised by MutL homologue 1 (*MLH1*) silencing and a high mutation burden in genes as *PIK3CA* and receptor tyrosine protein kinase (*ERBB3/HER3*). GS tumours show RAS homologue gene family, member A (*RHOA*) and E-cadherin (*CDH1*) gene mutations and the Claudin-18 (CDLN18)-Rho GTPase activating protein 6/26 (ARHGAP6/26) translocation. Finally, CIN can have *TP53* mutations and human epidermal growth factor receptor-2 (*HER2*) amplification in almost 25% of cases.

Claudins (CDLNs) are a family of at least 27 transmembrane proteins, first described by Tsukita et al.^6^ They are the major component of the tight junctions (TJ).^7,8^ TJ are the main cell–cell contacts among epithelium and endothelium.^9^ Particularly, CDLNs are composed by an extracellular loop, four4 transmembrane domains (including the N-terminus) and a cytoplasmic domain (including the C-terminus).^8^ The C-terminal domain exhibits binding domains for a complex of proteins such as the scaffolding zonula occludens proteins (ZO) ZO-1, ZO-2, ZO-3 and multi-PDZ domain protein 1 (MUPP1), which are involved in signalling pathways.^10^ CDLNs are mainly localised in the apical regions of the cellular membrane and play a critical role in cell–cell adhesion, maintenance of cell polarity and in selective paracellular permeability.^7–9^

Different CLDNs are expressed in various tissues^8^ and can be altered during carcinogenesis.^11^ For example, Claudin-6 is involved in breast cancer and in cervical cancer development,^12–14^ Claudin-11 in cutaneous melanoma,^15^ Claudin-1, -4, -7 in colorectal cancer^16^ and Claudin-8 in prostate cancer.^17,18^

Claudin-18 (CLDN18) is a highly specific TJ component of the stomach. It is expressed in foetal and in adult normal gastric mucosa.^11,19^ CLDN18 has two isoforms: Claudin-18.1 and Claudin-18.2, specific for pulmonary and gastric tissue, respectively. GCs and their metastases can retain the expression of this TJ protein.^8^

Targeted treatment of GC is in continuous evolution.^8,20–22^ Claudin 18.2 (CDLN18.2) represents an ideal therapeutic target due to its trans-membranous localisation. Claudiximab (IMAB362) is a monoclonal recombinant chimeric antibody (IgG1) specific for the CLDN18.2.^23^ The antibody can bind CLDN18.2 on the cellular surface and the binding induces the activation of antibody and complement-dependent cytotoxicity. IMAB362 is currently tested in several clinical trials for treatment of advanced gastric carcinomas alone or in combination with standard chemotherapy, showing a favourable safety profile and promising preliminary results in terms of clinical efficacy.^8,20^

With the present study, we investigated CDLN18 expression in 523 GCs and GECs, focusing on its association with the clinicopathological and molecular parameters.

## Materials and methods

### Case selection

A total of 523 archival cases of surgically treated GCs (*n* = 408) and GECs (*n* = 115) were retrieved from the archives of the Surgical Pathology Unit (Padua University). GECs were defined according to the American Joint Committee on Cancer (AJCC) TNM Classification Eighth edition, which define GECs as cancers with epicentre distant no more than 2 cm from the gastric cardia.^24^

All tissue samples were processed according to standard protocols, with formalin fixation time <48 h. All cases were jointly reassessed by 3 pathologists (R.C., I.C. and M.F.), and representative, non-necrotic cancer specimens were selected for tissue microarray (TMA) construction. When available (*n* = 135), metastatic nodes were also included in the analysis. For metastatic samples, only metastatic foci >2 mm with >40% neoplastic component were selected for TMA preparation.

Only material that was not required for diagnosis was used and all patients signed an informed consent approved by the University Hospital of Padua Review Board, which allows researchers to use excess material for research purposes. The study was approved by the local Ethic Committee.

### TMA construction

Two neoplastic areas from two separate formalin-fixed, paraffin-embedded (FFPE) blocks were selected and tissue cores (1 mm diameter) were punched out of these areas using the Tissue ArrayerMinicore 3 (Alphelys, Plaisir, France), as previously described.^21^

In 520 of the 523 primary GCs/GECs, 2 tissue cores were obtained from each selected area (i.e., 4 samples per tumour); in 3 of the 523 cases, only 1 cancer tissue core was available from each neoplastic area (i.e., 2 samples per tumour). From the 135 synchronous nodal metastases, 2 tissue cores were obtained in 115 cases, whereas a single tissue core was obtained from 20 metastatic nodes.

As a result, a total of 2086 tissue cores of primary GC/GECs and 250 tissue cores of metastatic nodes were collected in 34 TMA blocks (average number of cores 68.7 per block; range 40–128).

### Immunohistochemistry (IHC)

IHC stains were performed using the Bond Polymer Refine Detection Kit (Leica Biosystems, Newcastle upon Tyne, UK) on BOND-MAX automated IHC stainer (Leica Biosystems) and the UltraView DAB Detection Kit on Ventana Benchmark Ultra automated IHC staining system (Roche Diagnostics, Basel, Switzerland). Four-μm-thick FFPE sections were incubated with the primary antibodies for Claudin-18 (clone 34H14L15; Invitrogen, Carlsbad, CA; dilution 1:200), MSH6 (clone EP49; Agilent, Santa Clara, CA; dilution 1:25), PSM2 (EP51; Agilent; dilution 1:20), MLH1 (clone ES05; Agilent; dilution 1:25), MSH2 (FE11; Agilent; dilution 1:25), p53 (clone DO-7; Agilent; prediluted), p16INK4A (clone JC8; Santa Cruz Biotechnology, Dallas, TX; prediluted), PATHWAY HER2/neu (4B5) (Ventana Medical Systems, Roche Diagnostics) E-cadherin (clone NCH-38; Agilent; dilution 1:200), and PDL-1 (clone API3171 AA; Biocare, Pacheco, CA; prediluted).

For the evaluation of CLDN18, the membrane immunoreaction was assessed using a semi-quantitative pathology *H*-score, defined as the aggregate of total percentage of tumour cells expressing CLDN18 at each particular intensity level from 0, +1 (weak intensity), +2 (moderate intensity) or +3 (strong intensity). In brief, the *H*-score was defined as: (Percentage of CLDN18 1+ tumour cells multiplied by intensity of 1) + (Percentage of CLDN18 2+ tumour cells multiplied by intensity of 2) + (Percentage of CLDN18 3+ tumour cells multiplied by intensity of 3). Thus this composite score can range from 0 (a tumour which is completely negative) to a maximum of 300 (a tumour in which all the cells feature a 3+ staining). Scores were categorised in negative/low (0 = 0–50) and positive/high (1 = 51–300). Where present, nuclear and/or cytoplasmic CLDN18 expression was noted but not retained for scoring.

Deficient mismatch repair (MMRd) status was assessed by testing MSH2, MSH6, MLH1 and PSM2, and samples were defined as MMRd when one or both proteins resulted negative.^25^

p53 was considered as aberrant in the presence of complete loss or diffuse and strong nuclear immunostaining in neoplastic cells.^26^

For the evaluation of p16, immunoreaction was assessed using a four-tier classification: 0, complete absence of p16 staining in all neoplastic cells; 1, staining only in isolated and dispersed neoplastic cells; 2, staining in patchy and scattered clusters of neoplastic cells; and 3, dense and continuous cytoplasmic/nuclear staining in all neoplastic cells (1). The resulting values were combined in 2-point total scale, characterised by negative/low (0 and 1) and positive/high (2 and 3) expression.

For the evaluation of HER2, the four-tier modified Herceptest score for biopsies was used. Score 0/1+ (no membranous immunostaining in any neoplastic cells/presence of tumour cell cluster with barely perceptible membranous reactivity irrespective of percentage of tumour cells stained) as negative; 2+ (presence of tumour cell cluster with weak or moderate basolateral–lateral membranous reactivity irrespective of the percentage of tumour cells stained) as equivocal; and 3+ (presence of tumour cell cluster with strong complete, basolateral or lateral membranous reactivity irrespective of the percentage of tumour cells stained) as positive.^21^

E-cadherin expression was considered altered in the presence of complete loss or markedly reduced membranous staining (>30%), regardless of nuclear/cytoplasmic staining.^26^

Only tumour PD-L1 expression was retained for scoring, and a 1% cut-off was used in the analysis.

### HER2 chromogenic in situ hybridisation (CISH)

CISH was performed according to the manufacturer’s protocol (Dako Her2 CISH pharmDx Kit; Dako, Glostrup, Denmark). Areas with the highest *HER2* counts with non-overlapping nuclei were analysed by counting *HER2* and centromeric probe 17 (*CEP17*) signals in at least 40 nuclei. The ratio *HER2/CEP17* was calculated. A case was considered *HER2* amplified when the signal ratio was ≥2.0 or when *HER2* signal cluster was observed.

### EBER in situ hybridisation (ISH)

The Bond ready-to-use ISH EBER Probe was used in a Leica Bond-Max automation system according to the manufacturer’s instructions (Leica Biosystems) to detect EBV infection.

### Statistical analysis

Differences and associations between CLDN18 and clinicopathological variables or other IHC markers were tested by applying the χ^2^ test and Fisher Exact test. A *p* value <0.05 was considered significant.

## Results

### Clinicopathological findings

Overall, the mean age of the patients was 69.3 ± 12.4 years (median 60; range 25–95). The male-to-female ratio was 1.98 (Table 1). All patients were Caucasian.

**Table 1:** Clinicopathological features of the considered series according to CLDN18 status

Variables	*n* (na)	CLDN18 negative (%)	CLDN18 positive (%)	*p*
Age (years), mean ± SD	523 (13)	69.3 ± 12.4	69.5 ± 12.0	NS
*Gender*				NS
Male	348 (7)	237 (69.5)	104 (30.5)	
Female	175 (6)	123 (72.8)	46 (27.2)	
*Localisation*				NS
Stomach	408 (11)	279 (70.3)	118 (29.7)	
GEC	115 (2)	81 (71.7)	32 (28.3%)	
*Gastric localisation*				*p* = 0.016
Antrum	198 (4)	145 (74.7)	49 (25.3)	
Corpus	191 (3)	119 (63.3)	69 (36.7)	
*Tumour grading*				NS
1	71 (3)	51 (75.0)	17 (25.0)	
2	204 (4)	141 (70.5)	59 (29.5)	
3	248 (6)	168 (69.4)	74 (30.6)	
*TNM staging*				NS
I	150 (4)	115 (78.8)	31 (21.2)	
II	189 (4)	125 (67.6)	60 (32.4)	
III	139 (3)	92 (67.6)	44 (32.4)	
IV	45 (2)	29 (67.4)	14 (32.6)	
*Lauren histotype*				*p* = 0.009
Intestinal	291 (7)	202 (71.1)	82 (28.9)	
Diffuse	117 (4)	65 (57.5)	48 (42.5)	
*Ming histotype*				NS
Infiltrative	339 (9)	217 (65.8)	113 (34.2)	
Expanding	69 (2)	50 (74.6)	17 (25.4)	

*n* number of cases, *na* data not available for CLDN18 expression, *GEC* gastro-oesophageal carcinoma, *NS* not significant, *CLDN18* Claudin-18, *TNM* tumour, node, metastasis

Among GCs, 198 (48.5%) were localised in the antrum, 191 (46.8%) in the corpus, 10 (2.5%) cases were categorised as *linitis plastica* and 9 cases (1.6%) were recurrent disease at the gastro-duodenal/jejunal anastomosis. The GECs were 115. The metastatic samples were 135, 112 (82.9%) from gastric and 23 (17.1%) from gastro-oesophageal tumours.

According to the 2010 World Health Organisation criteria, 328 (80.3%) and 112 (97.3%) cases were NOS (not otherwise specified) adenocarcinomas, of the stomach and of the gastro-oesophageal junction, respectively. Among the gastric adenocarcinomas, 1 (0.2%) was hepatoid, 14 (3.4%) were mucinous, 2 (0.5%) were papillary, 27 (6.6%) were tubular, 2 (0.5%) had papillary and tubular features and 34 (8.5%) were poorly cohesive carcinomas (including signet-ring type). Among the GECs, 1 (1%) was an adenosquamous carcinoma and 2 (1.7%) were poorly cohesive carcinomas, signet-ring type.

When evaluating the grade of differentiation, 71 cases (13.5%) were G1, 204 cases (39%) were G2 and 248 cases (47.5%) were G3. According to tumour staging, 150 cases (28.7%) had stage I (IA, 63; IB, 87, respectively); 189 cases (36.1%) had II stage (IIA, 106; IIB, 83, respectively); 139 cases (26.6%) had III stage (IIIA, 72; IIIB, 25; IIIC, 43, respectively); 45 cases (8.6%) had IV stage.

In GCs, 117 cases (28.6%) were of Lauren diffuse type, and 291 cases (71.4%) were of intestinal type. According to Ming Classification, 69 cases (16.9%) were expanding, and 339 cases (83.1%) infiltrative.

### CLDN18 expression: prevalence and clinicopathological associations

**Fig. 1:**
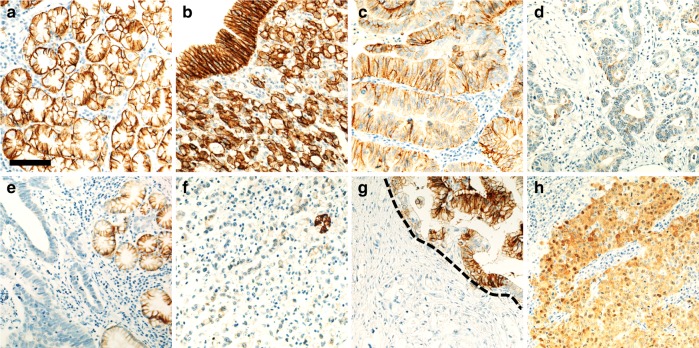
**a** CLDN18 expression in normal gastric glands. **b** High CDLN18 expression in a gastric cancer (the adenocarcinoma is covered by normal gastric glands, which show an intense CLD18 positivity). **c** Moderate CLDN18 expression in cancer glands. **d** Low CLDN18 expression. **e** A negative CDLN18 tumour near to positive CLDN18 normal gastric glands. **f** Low CLDN18 expression in signet-ring cell carcinoma surrounding a positive single normal gastric gland. **g** CDLN18 intratumour variability. **h** Moderate-to-strong nuclear and cytoplasmic CLDN18 positivity. (Original magnifications ×20; scale bar 100 µm)

CLDN18 moderate-to-strong membranous expression was always observed in non-neoplastic gastric mucosa (Fig. 1). In cancer cells, CLDN18 was considered as positive only if membranous staining was present. Overall, any CLDN18 expression was present in the 61.6% (327/510) of primary cases and in 55.3% (73/132) of nodal metastases. High CLDN18 expression (i.e. *H*-score >51) was present in the 29.3% (150/510) of primary cases and in 34.1% (45/132) of nodal metastases (Fig. 1). In 108/510 (21.2%) primary tumours, only a weak (i.e. 1+) CLDN18 expression was observed, and only 26 (5.1%) of these cases were classified as high CLDN18 tumours. Moderate (i.e. 2+) membranous staining was observed in 101/510 (19.8%), whereas strong CLDN18 expression (i.e. 3+) was observed in 38/510 (7.5%). Among metastatic samples, 15/132 (11.4%) showed only a weak CLDN18 membranous staining, 32/132 (24.2%) were characterised by a moderate expression, whereas 11/132 (8.3%) showed a strong membranous expression.

**Fig. 2:**
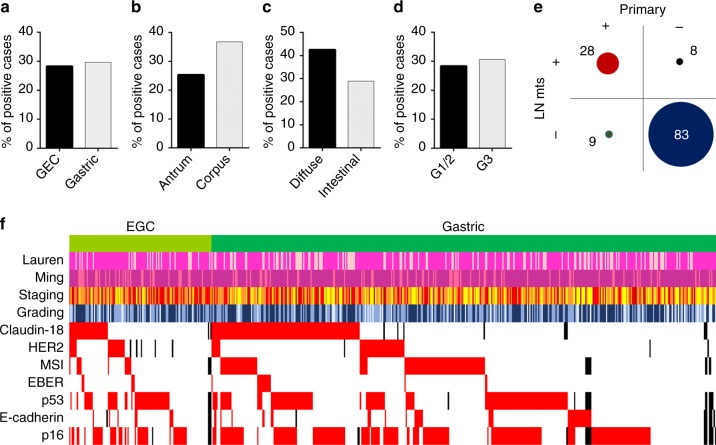
Distribution of CLDN18-positive primary cases: among gastro-oesophageal (GEC) and gastric (GC) carcinomas (**a**), according to location within the gastric mucosa (**b**), according to tumour histotypes (**c**) and according to tumour grading (**d**). **e** CLDN18 status assessed in matched primary and metastatic tumours (*n* = 128). **f** Graphic summary of clinicopathological and immunohistochemical/in situ hybridisation results observed in the present study. Cases are disposed in columns, clinicopathological and molecular features in rows; missing data are in black, positive data are in red (i.e. high-CLDN18 expression; HER2 overexpression or amplification; presence of alterations in the proteins of the DNA mismatch repair; EBER-positive staining; p53 aberrant cases; E-cadherin-negative cases; p16-negative cases). Lauren lighter lines: diffuse-type carcinomas; Ming lighter lines are cases with expansive growth pattern; tumour staging from yellow (stage I) to dark red (stage IV); tumour grading from light blue (grade 1) to dark blue (grade 3)

High CLDN18 was associated with tumour site (antrum vs corpus), with a higher prevalence of positive cases among proximal (i.e. corpus) GCs (*p* = 0.016), whereas the distribution between GCs and GECs showed no statistically significance (29.7 vs 28.3%). High CLDN18 expression was also associated with the Lauren Classification, with higher prevalence of positive cases in the diffuse pattern (*p* = 0.019) (Fig. 2a–d). No association emerged between CLDN18 and age/sex, grading, staging and Ming Classification. We tested the prognostic impact of CLDN18 on the series of 45 stage IV cases, in which 10 (22.2%) tumours showed high CLDN18 expression, but the association between CLDN18 status and patients’ prognosis was not significant in this small series of advanced cancers.

Of note, a strong nuclear and/or cytoplasmic positivity was observed in 115 primary cases (22.5%): 95 with a nuclear positivity, 13 with a cytoplasmic positivity and 7 with a mixed nuclear/cytoplasmic positivity. In 47/95 (49.5%) cases with nuclear staining, a concurrent membranous positivity was observed and only 10 of them were categorised as high CLDN18 tumours. Similarly, among metastatic samples, 27 (20.5%) had a nuclear positivity, 6 (4.5%) a cytoplasmic positivity and 5 (3.8%) a mixed positivity. The nuclear and/or cytoplasmic positivity showed no statistically significant associations with tumour location. Furthermore, no significant associations emerged comparing nuclear/cytoplasmic with membranous CLDN18 expression and with pathological staging.

Intratumoural variability of membranous CLDN18 expression was investigated, considering CLDN18 expression among the multiple TMA cores collected from different areas of the same tumour. A tumour was considered as CLDN18 heterogeneous in case of concomitant presence of high-CLDN18 and low-CLDN18 TMA cores. Among primary tumours, 160 GCs (40.3%) and 38 GECs (33.6%) showed intratumoural variability within the analysed cores. Similar results were observed for metastatic samples, with a total of 38 cases (28.8%) with heterogeneous CLDN18 status. Focussing on CLDN18 expression, as assessed in matched primary and metastatic samples, this analysis was possible for 128 couples. CLDN18 status was consistent between the two matched samples in 111 cases (86.7%; 83 negative and 28 positive cases), with only 17 cases showing CLDN18 discordant status (Fig. 2e).

### CLDN18 expression according to the IHC profiling of the tumours

Among primary tumours, 318/507 cases (62.7%) showed a normal p53 immunoreactivity, and 239/503 (47.5%) were p16 negative. Similar results were observed for metastatic lesions, with 79/119 cases (66.4%) characterised by the absence of p53 alterations and 62/119 cases (52.1%) resulting as p16 negative. Only 51/511 (10.0%) primary neoplasms showed completely loss of E-cadherin membranous expression and similar results were obtained in nodal metastasis, in which 17/125 cases (13.6%) were characterised by E-cadherin loss. A total of 63/515 (12.2%) primary adenocarcinomas showed a HER2 overexpression or *HER2* gene amplification. In detail, 43 were gastric tumours (10.6% of HER2 alterations), and 20 were gastro-oesophageal tumours (18.2% of HER2 alterations). MMRd was observed in 113/511 cases (22.1%) of primaries and 19/119 (16.0%) of nodal metastases. No associations emerged between CLDN18 expression and p53, p16, E-cadherin, HER2 or MMRd in both primary tumours and in metastatic nodes (Tables 2 and 3 and Fig. 2).

**Table 2:** Association between CLDN18 status and the other biomarkers in primary tumours (NS = not significant)

Biomarker	CLDN18 negative (%)	CLDN18 positive (%)	*p*
*EBER*			*p* < 0.001
Positive	6 (30.0)	14 (70.0)	
Negative	348 (71.0)	142 (29.0)	
*p53*			NS
Aberrant	130 (68.8)	59 (31.2)	
Normal	220 (69.2)	98 (30.8)	
*p16*			NS
Positive	177 (74.1)	87 (25.9)	
Negative	172 (65.2)	68 (34.8)	
*MMR*			NS
Deficient	75 (66.4)	38 (33.6)	
Proficient	273 (68.8)	124 (31.2)	
*E-cadherin*			NS
Positive	323 (70.4)	136 (29.6)	
Negative	41 (80.4)	10 (19.6)	
*HER2*			NS
Amplified	48 (76.2)	15 (23.8)	
Not amplified	321 (71.8)	126 (28.2)	

*CLDN18* Claudin-18

**Table 3:** Association between CLDN18 status and the other biomarkers in metastatic tumours (NS = not significant)

Biomarker	CLDN18 negative (%)	CLDN18 positive (%)	*p*
*EBER*			NE
Positive	0 (0.0)	0 (0.0)	
Negative	92 (69.7)	40 (30.3)	
*p53*			NS
Aberrant	30 (75.0)	10 (25.0)	
Normal	56 (70.9)	23 (29.1)	
*p16*			NS
Positive	41 (71.9)	26 (28.1)	
Negative	42 (67.7)	19 (32.3)	
*MMR*			NS
Deficient	13 (68.4)	6 (31.6)	
Proficient	75 (75.0)	25 (25.0)	
*E-cadherin*			NS
Positive	75 (69.4)	33 (30.6)	
Negative	12 (70.6)	5 (29.4)	
*HER2*			NE
Amplified	0 (0.0)	0 (0.0)	
Not amplified	90 (71.4)	36 (28.6)	

*CLDN18* Claudin-18; *NE* not evaluable

A total of 20/523 (3.8%) tumours were positive for EBER ISH. Of this group, 15 were GCs and 5 GECs. A significant association was observed between EBER status and CLDN18 expression (*p* < 0.001). In fact, 14/20 (70%) of EBER-positive tumours showed high expression of CLDN18. Moreover, 4 further EBER-positive cases showed low (i.e. >1 and <51) CLDN18 expression. Only two EBER-positive cases did not show any CLDN18 expression. The mean age of EBER positive tumours was 64.6 years, and the male-to-female ratio was 3:1. Among the 15 EBV-associated GCs, 12 were located in the gastric corpus, 2 in the antrum, and 1 in an oesophago-jejunal anastomosis.

Owing to material exhaustion, only 210 cases were tested for PD-L1 expression, of which 79 with nodal metastases. Considering the neoplastic PD-L1 expression, 18/184 GC cases (9.8%) were positive, whereas only 1/26 (3.8%) GEC showed at least a 1% positivity prevalence within cancer cells. On the other hand, considering positive a case with a Combined Positive Score (CPS) of ≥1%, 41/184 (22.3%) GC and 4/26 (15.4%) GEC resulted PD-L1 positive. PD-L1-positive cases showed a trend towards a higher prevalence of EBER positivity (6.7% vs 3.0%), MMRd tumours (31.1% vs 22.4%) and CLDN18 high expression (38.8% vs 29.7%). Similar results were observed in the metastatic samples. The trend was more pronounced for the GC group, in which PD-L1-positive cases showed a higher prevalence of EBER-positive cases (7.3%), MMRd tumours (34.1%) and CLDN18 high expression (41.5%).

Immunohistochemical profiling has recently emerged as a suitable alternative for molecular classification of GC.^26,27^ We focussed on the work of Ahn and collaborators^26^ to IHC categorise our series according to TCGA and the Asian Cancer Research Group (ACRG) molecular classifications of GC. According to TCGA molecular subtypes, 20 cases were EBV related (14 CLDN18 high; 70.0%), 77 cases were MSI (23 CLDN18 high; 29.9%), 36 cases were GS (5 CLDN18 high; 13.9%) and 366 cases were of the CIN subtype (107 CLDN18 high; 29.2%). According to ACRG molecular subtypes, 77 cases were MSI (23 CLDN18 high; 29.9%), 38 cases were MSS/EMT (7 CLDN18 high; 18.4%), 224 cases were MSS/p53+ (71 CLDN18 high; 31.7%) and 159 cases were MSS/p53− (48 CLDN18 high; 30.2%). Thus EBV-associated cancers showed the highest prevalence of CLDN18-positive tumours, whereas the GS and MSS/EMT subgroups showed the lowest prevalence in CLDN18-positive status.

## Discussion

The main aim of this study was to investigate CLDN18 expression in a large mono-Institutional series of GCs and GECs using IHC. Taking into account the emergent role of the monoclonal antibody Claudiximab (IMAB362) targeted against the isoform Claudin-18.2, these results were compared to clinicopathological and molecular parameters, in order to detect possible distinctive features of CLDN18-positive GC/GECs.

CLDN18 is a highly specific gastric claudin expressed in the normal adult gastric mucosa, as well in the gastric foetal tissue, with a tendency to be conserved in GCs.^11,19,28^ Our results are consistent with previous findings, since the presence of CLDN18 was documented in 29.4% primary cases and in 34.1% nodal metastases. No significant differences emerged in relation to patients’ age, sex, gastric vs gastro-oesophageal tumour localisation, grading, pathological staging, and Ming Classification. Among the GCs, we found a significant association between CLDN18 expression and gastric tumour localisation (i.e. tumour localised in the gastric corpus showed a higher prevalence of CLDN18-positive cases) and Lauren Classification. These data could be explained by both the role of CLDN18 in the paracellular ion transport, which is primarily associated with the gastric body^29^ and by the “intestinalisation” observed during Correa’s cascade in most antral GCs, which can lead in many instances to the loss of gastric-specific markers.^19^

According to the Lauren Classification, a higher prevalence of positive CLDN18 cases had a diffuse type. Considering the TGCA GC classification, GS tumour category often shows a prevalent diffuse pattern. A typical molecular alteration of this subtype is an interchromosomal translocation between *CLDN18* gene and *ARHGAP26*. ARHGAP26 is a GTPase-activating protein (GAP). Its role is to facilitate RHO GTPase to the GDP state and consequently to induce the cellular motility.^2^ These types of translocations are common in diffuse GC and have been demonstrated to be negative prognostic factors.^30–33^ Of note, Tanaka and colleagues demonstrated that cases with *CLDN18* translocation are significantly characterised by a CLDN18 IHC overexpression.^32^

An interesting observation in our study was the nuclear and/or cytoplasmic CLDN18 immunoreactivity. This intracellular distribution was not significantly linked to a loss or weaker expression of membranous localisation. Previous studies have already focussed on the TJ nuclear/cytoplasmic positivity.^34,35^ Somoracz and colleagues^34^ demonstrated a nuclear positivity of tricellulin in a subset of hepatocellular carcinoma. The nuclear positivity was associated with a weaker membranous expression of this TJ, suggesting a possible disturbed intracellular trafficking of molecules. French et al.^35^ stressed that the nuclear Claudin-1 (CLDN1) was linked to benign nevi and to early melanomas vs a high cytoplasmic and membranous CLDN1 expression mainly linked to the metastatic counterparts. Furthermore, these authors demonstrated that CLDN1 translocation from nucleus to cytoplasm was driven by protein kinase A (PKA) via phosphorylation.

Among the CLDN18-positive cases, another point was the high prevalence of its membranous intratumoural variability. This has been similarly demonstrated for HER2 in GC^36–40^ and could affect (i) biomarker evaluation in biopsy specimens and (ii) any targeted therapeutic approach. Further “real world” studies should investigate the minimum number of GC/GEC biopsies to have an adequate CLDN18 evaluation.

The association between CLDN18 expression and p53, p16, E-cadherin, PD-L1 and HER2 was investigated. The only significant association emerged between CLDN18 and E-cadherin. Higher prevalence of positive E-cadherin was found among positive CLDN18 tumours. This association was independent of the Lauren Classification.

We found a significant association between CLDN18 and EBV-positive status. Shinozaki et al.^3^ previously investigated the Claudins’ expression in EBV-GCs compared to EBV-negative GCs and their results indicated a CLDN18-positive predominance in EBV-associated GCs. Since preserved expression of CLDN18 was described not only in mature but also in immature gastric epithelium,^19^ our data could support the hypothesis that EBV-associated GCs could arise directly from immature proliferating cells. There were no significant differences between prevalence of EBV-associated GCs and EBV-positive GECs. Jovov et al.^41^ demonstrated an almost absolute predominance of CLDN18 expression in Barrett’s oesophagus compared to other Claudins. Shinozaki’s study^3^ underlined the lack of CLDN18 in intestinal metaplasia in the stomach. These previous results support the hypothesis that CLDN18 expression is mainly dependent on the molecular profile of immature tissue-specific cells regardless of the presence or not of an intestinal phenotype. Anyway, this point remains unclear and would require further investigation.

The prevalence of CLDN18-positive cases is relatively lower in our study in comparison to the recent published clinical trial^42^ or a previous paper by Matsusaka and colleagues;^28^ however, our series considered only a 8.6% of stage IV tumours and this can significantly affect the prevalence of CLDN18-positive cases. Moreover, different antibodies are available, and they are characterised by different sensitivities/specificities to CLDN18. In particular, by checking the Human Protein Atlas database (www.proteinatlas.org; 21 January 2018) three different antibodies are on the market and detect, on the same series of GC specimens, CLDN18 high expression in 36.4 to 70.0% of the cases, further supporting the need for a standardisation of testing.

In summary, this is the largest study investigating CLDN18 expression among GECs. One third of the cases retained CLDN18 expression and this was significantly associated with gastric corpus location, diffuse-type GC and with the presence of EBV infection. Our group has recently started a new prospectively conceived translational study on a selected series of stage IV GC, focusing on CLDN18.2 isoform and its prognostic role and predictive significance to current standard treatments.

## Data Availability

The data sets used and/or analysed during the current study are available from the corresponding author on reasonable request.
